# Polarizer-assisted pupillometry through closed eyelids, overcoming pupil position dependence

**DOI:** 10.1117/1.JBO.31.6.064303

**Published:** 2026-02-12

**Authors:** Michal Tepper, Omer Ben Barak-Dror, David Haggiag, Israel Gannot, Yuval Nir

**Affiliations:** a Tel Aviv University, School of Biomedical Engineering, Faculty of Engineering, Tel Aviv, Israel; b Tel Aviv University, Department of Physiology and Pharmacology, Faculty of Medicine, Tel Aviv, Israel; c Johns Hopkins University, School of Medicine, Baltimore, Maryland, United States; d Tel Aviv University, Sagol School of Neuroscience, Tel Aviv, Israel

**Keywords:** optical imaging, polarization, pupillometry, sedation, sleep

## Abstract

**Significance:**

Accurate monitoring of pupil size and gaze direction is critical in clinical and research contexts; however, current pupillometry methods require open eyes, limiting their use in patients under anesthesia, sedation, or sleep. Short-wave infrared (SWIR) imaging enables noninvasive closed-eye pupillometry, but challenges remain due to eyelid glare, gaze variability, and low signal-to-noise ratio (SNR).

**Aim:**

We aimed to enhance closed-eye pupillometry by integrating polarization filters into the SWIR imaging system and developing improved algorithms for pupil localization and gaze direction estimation under natural closed-eye conditions.

**Approach:**

Experiments were conducted on healthy volunteers using SWIR imaging with different polarizer configurations (parallel, partially crossed, crossed, and no polarizers). Pupillary light reflexes (PLR) were recorded under open- and closed-eye conditions with both forward fixation and varying gaze directions. Image analysis incorporated brightness difference imaging and statistical modeling to evaluate maximal brightness change and SNR.

**Results:**

In open-eye settings, parallel polarizers produced the strongest PLR signal, but in closed-eye conditions, crossed polarizers significantly improved image quality by suppressing eyelid glare. The crossed configuration yielded the highest PLR brightness change and SNR compared with parallel or no polarizers, enabling reliable pupil localization across multiple gaze directions. Improved algorithms allowed robust PLR detection even under natural eyelid closure and variable gaze positions.

**Conclusions:**

Integrating crossed polarizers into SWIR-based pupillometry substantially enhances signal fidelity and pupil localization through closed eyelids. This approach overcomes major limitations of previous methods and enables accurate, touchless pupillometry in clinically relevant conditions. These advances pave the way for applications in anesthesiology, sleep medicine, and neurocritical care.

## 1 Introduction

Pupillometry, the measurement of pupil size and reactivity, is a valuable tool in various fields, including neuroscience, cognitive research, and clinical medicine. The muscles controlling pupil diameter are regulated by the autonomic nervous system, causing pupil constriction and dilatation. Pupil dynamics occur in response to changes in ambient light, fixation, following transient external stimuli, such as light or sound, activation of neuromodulatory pathways, such as noradrenaline signaling,^1^^–^^3^ or due to internal factors, including arousal, pain, cognition, and mental effort.^4^^–^^6^

Modern pupillometry methods are used for a variety of clinical applications and have evolved from manual measurement using a penlight and a ruler to automated devices measuring and tracking pupil size and position over time.^7^ In medicine, pupillometry is used as a noninvasive tool to detect abnormalities in autonomic function, which can indicate issues such as traumatic brain injury,^8^ stroke,^9^ intracranial pressure,^10^ neurodegenerative diseases,^11^ diabetes complications,^12^ and brainstem function impairment.^7^^,^^13^^,^^14^ Given its tight link with arousal, pupil size also can be a useful marker for anesthesia depth^15^^,^^16^ and sleep stages.^17^^,^^18^ In psychology, pupillometry is used to study emotional and cognitive responses, enhancing the study of human behavior and mental processes.^5^^,^^19^^,^^20^ The correlation of pupil dynamics to internal cognitive and physical processes makes it a valuable tool for biofeedback applications,^21^^,^^22^ as well as an additional input source for brain–computer interface systems.^23^^,^^24^

Despite the ongoing research and development of quantitative automated techniques and methods, clinical pupillometry requires a human operator, making the measurements time-consuming, intermittent, and less accurate.^25^ Manual measurements, as well as automated devices, rely on viewing the pupil in visible or near-infrared (NIR) wavelengths. As the eyelid is relatively opaque in these wavelengths, these pupillometry devices can only be used in open-eye situations. Some attempts have been made in closed-eye pupillometry in specific species, such as pigeons, where the eyelid is translucent,^26^ or in humans when delivering light through the temple to “back-illuminate” the eye, but this technique has limited applicability as it requires frequent breaks to avoid heat damage to the tissue.^27^ Thus, despite its clinical potential, pupillometry in its current form cannot be employed to continuously monitor closed-eye situations such as surgical anesthesia or sleep.

We recently presented a method enabling continuous touchless pupillometry in closed eyes using short-wave infrared (SWIR) imaging and dedicated algorithms.^28^ As SWIR wavelengths can penetrate through the eyelid skin, SWIR imaging can provide visualization of the iris and pupil behind it.^29^ The original proof-of-concept monitored gaze direction and pupil size changes induced by the pupillary light reflex (PLR) in highly controlled settings when participants directed their gaze forward and held one eye closed. To move toward clinical bedside applications, we opted to track pupil size, whereas eye gaze and pupil position change freely when both eyes are naturally closed, and we aimed to increase its performance and improve its signal-to-noise ratio (SNR). One of the main contributors to the noise in the SWIR image of the closed eye is the reflected light, or glare, from the eyelid skin surface. Eliminating this glare is desirable to improve the visualization of light scattered from the pupil and iris.

One of the most widely used optical techniques to improve tissue visualization is polarization imaging. It uses polarizers, which are optical elements that selectively transmit light waves with a specific polarization orientation while blocking others. By selectively filtering polarized light, polarizers aid in visualizing structures with greater clarity and detail. When integrated into optical devices, polarizers can improve diagnostic accuracy and facilitate insights into deeper biological processes.^30^ In biomedical imaging, polarizers play a critical role in enhancing contrast, reducing glare, and improving image quality in modalities such as microscopy,^31^ optical coherence tomography,^32^ biomedical polarization imaging,^33^ and fluorescence imaging.^34^ As the polarization of reflected or emitted light depends on the structure of the observed object, the use of polarizers enables researchers to selectively enhance or minimize the visibility of certain features.

In addition, the use of a crossed-orientation polarizer configuration (one polarizer between the illumination source and the tissue, and another polarizer with orthogonal orientation placed between the tissue and the camera) can assist in removing surface glare from the illuminated tissue. When the tissue is illuminated, the light is partially reflected from the tissue surface while relatively maintaining the original polarization. Most of the incident light does not undergo surface reflection but rather penetrates the tissue, where it is subjected to multiple scattering events by heterogeneous structures. These interactions result in depolarization, such that the emerging light exhibits a randomized polarization distribution. As a result, the second polarizer placed before the detector transmits approximately half of the depolarized light, thus permitting meaningful image formation even in a crossed setup while suppressing a substantial portion of the surface glare.

When imaging the eye, polarized imaging of the iris and cornea assists clinicians in gathering valuable information about tissue composition and pathological changes, aiding in the early detection and monitoring of diseases such as glaucoma or malaria.^35^^–^^39^ Polarimetric imaging of the eye is influenced by the birefringence properties of the iris, which alter the polarization of transmitted light. This variability complicates the determination of the optimal polarizer configuration for polarization-based eye imaging devices.^40^^,^^41^ Although polarizers could potentially reduce the noise and improve the accuracy of closed-eye pupillometry, they also reduce signal amplitude as they decrease light intensity reaching the camera. Thus, a detailed examination is necessary to determine if and how polarization imaging could improve closed-eye pupillometry and gaze direction estimation. To this end, we investigated polarized imaging in open-eye settings to inform subsequent experiments with closed eyes. The study also implemented further improvements to closed-eye imaging via automatic identification of pupil position in closed eyes that enables pupillometry in the face of ongoing eye movements and by transitioning to naturally closed-eye settings.

## 2 Methods

### 2.1 Study Design

Experiments were conducted on healthy volunteers in accordance with the institutional review board of the ethical committee of Tel Aviv University (approval no. 0005827-1) and radiation safety requirements (IEC 62471 international standard). Each volunteer signed an informed consent form for participating in the study. The experiments were based on artificially inducing changes in pupil size (in response to brief light stimuli) and pupil position (directing gaze to specific directions in response to instructions) and measuring the PLR in closed-eye settings using the methods described below.

### 2.2 Experimental Setup

**Fig. 1:**
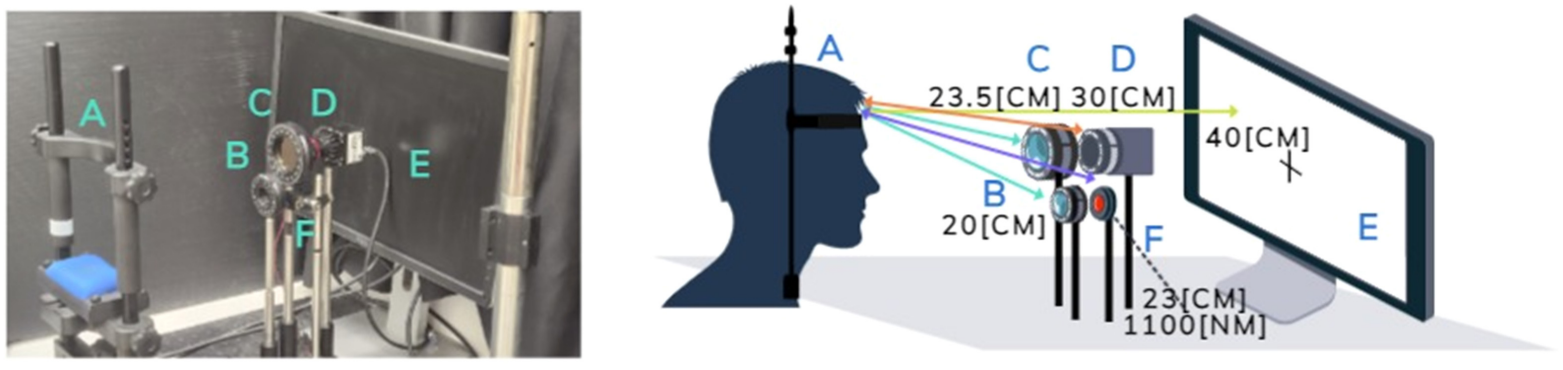
Experimental setup. (A) Chinrest, (B) LED polarizer, (C) camera polarizer, (D) camera, (E) screen, and (F) LED. The polarizers were replaced by a beam splitter at the position of C for the control configuration.

All experiments were conducted in a dark room. The experimental setup is shown in Fig. 1. Participants sat, whereas their head rested on a dedicated chinrest (Eyelink^®^, SR Research, Ottawa, Canada) with their heads facing a computer screen at a distance of 40 cm (Dell 27 Monitor—P2719H, typical brightness of 300 lux, Dell Inc., Round Rock, Texas, United States). They were asked to sit still and keep their eyes closed during the experiments, maintaining the same gaze direction unless requested otherwise, and were allowed at least 30 s to adapt to the dark before visual stimulation began. The participant’s face and eyes were illuminated with an 1100 nm light-emitting diode (LED) [Thorlabs, Newton, New Jersey, United States, M1100D1, 168 mW (min)] placed 23 cm from the chinrest at the approximate height of the eyes. Both eyes were imaged using a SWIR camera [WiDy SenS 640V-ST, New Imaging Technologies (NIT), Verrières le Buisson, France] equipped with a 25 mm lens (LM25HC, Kowa, Dusseldorf, Germany) placed 30cm from the chinrest directly in front of the participants at the approximate height of the eyes. The video was captured using the camera’s software, at a 31 Hz frame rate with a 15 ms integration interval. The main experimental setup included two polarizers: one in front of the LED (referenced in this paper as the “LED polarizer”) and the other in front of the camera (the “camera polarizer”) (W436160 and W428316, respectively, Thorlabs, Newton, New Jersey, United States).

To compare the results with a setting using unpolarized light while maintaining the same illumination intensity reaching the eyes, a control experimental setup was used. In this configuration, both polarizers were removed, and a beam splitter (CM1-BS014, Thorlabs) was placed in front of the illumination LED. The beam splitter reduced the light intensity by 50%, ensuring the eyes were exposed to the same illumination intensity in all three configurations, under the assumption of polarization-independent LED illumination.

### 2.3 Experimental Design

#### 2.3.1 Pupillary light reflex

Comparing different polarizer orientations is complicated by the different characteristics of each participant and experiment. Small changes in the participant’s position or orientation toward the camera may lead to different illumination levels on the eyelid. Therefore, standardized stimulation was used to induce comparable responses among different polarizer configurations. In each experiment, 10 PLR responses were induced by presenting a white screen stimulus for 2 s on the computer screen in front of the participants. Visual stimulations were interleaved by periods of dark inter-stimulus intervals lasting 20±2  s (to avoid prediction bias), as previously described.^28^ The PLR response is a reflexive response with a characteristic time course^42^ that is relatively consistent among experiments and could thus provide a reliable basis for comparison.

#### 2.3.2 Experiment 1—open eyes

We compared the PLRs in open-eye settings in three different polarizer orientations: parallel orientation (both polarizers horizontally aligned), partially crossed orientation (horizontal alignment for the LED polarizer and 45 deg orientation for the camera polarizer), and crossed orientation (horizontal alignment for the LED polarizer and vertical orientation for the camera polarizer). The LED polarizer orientation was horizontal in all experiments as it seemed to minimize the observed reflection from the open-eyelid edges.

#### 2.3.3 Experiment 2—forward-fixated PLR in closed eyes

The PLR experiment was conducted on six participants for each of the three configurations. The experiments were conducted using the control configuration (beam splitter without polarizers) and two polarizer configurations (in which the LED polarizer was oriented to block horizontally polarized light and the camera LED was set to either a horizontal or vertical orientation). Participants were asked to close both eyes and try to look forward (toward the estimated direction of the camera) to maintain a similar and central position of the pupil. The experiment lasted a total of ±240  s for each configuration.

#### 2.3.4 Experiment 3—PLR with varying gaze directions in closed eyes

A modified PLR experiment was conducted in which the participant’s gaze was not fixated directly at the center of the camera but otherwise was arranged as the forward-fixated experiment. Here, after a 15 s baseline period, participants were instructed to direct their gaze toward nine different directions, in set intervals, while their eyes were closed. The nine directions represented a 3×3 grid that included combinations of up-forward-bottom with left-center-right. For each direction, the participants were asked to keep their gaze fixed for 22 s, which included 10 s adaptation to the new gaze direction, 2 s light stimulation using the white screen, and a 10 s darkness period. Nonuniformity in timing among PLR instances was naturally introduced due to the use of verbal instructions and the need to change gaze directions. Therefore, jitter was not incorporated into the experimental design to simplify the experimental procedure.

### 2.4 Data Analysis

#### 2.4.1 Analysis of closed-eye images

PLR dynamics in closed-eye SWIR data was analyzed using the previously described ‘fixed circle’ approach.^28^ In this method, the average pixel intensity is calculated within a fixed circle around the estimated pupil center with an approximate diameter of the iris. When the pupil constricts or dilates, its dark pixels occupy a different portion of the fixed circle, affecting the average pixel intensity. Accordingly, we previously established that the dynamics of the intensity changes over time is similar to the changes in pupil size, facilitating the closed-eye estimation of pupil size. To ensure that changes in pixel intensity were not a result of external brightness changes, the average intensity within a control circle on a neutral area was calculated as a control. We used a white sticker placed on the participant’s forehead for the control analysis to account for brightness changes due to the participant’s movement while avoiding reflection fluctuations due to involuntary forehead muscle twitches that may occur in response to light stimulation.

For each frame of the captured video, we defined three circles: one for each eye and one as a control. Average pixel intensity data were forward–backward filtered, using a 1 Hz low-pass filter (Python function “Filtfilt”^43^—zero phase digital filter forward and backward). After this pre-processing, time courses were averaged for each participant to reflect their overall dynamics. Baseline brightness was defined as the average brightness during 2 s before light stimulation onset. Intensity dynamics were expressed in terms of pixel “darkness” changes (pixel brightness change in percentages multiplied by −1) to achieve a time course similar to pupil size changes (the average pixel “darkness” decreases when the pupil diameter decreases shortly after light stimulation, and vice versa).

Polarizer configurations were compared using two metrics that quantify changes in brightness relative to a baseline period, defined as the 2 s preceding light stimulus onset: (1) The maximum relative change in brightness from the average baseline level, divided by the baseline brightness value, and (2) the SNR, calculated as the maximum brightness change divided by the standard deviation of the baseline brightness. These metrics were chosen for their robustness and relative insensitivity to confounding factors such as participant movement and baseline pupil size variability.

#### 2.4.2 Analysis of open-eye images

The analysis method used for the closed eyes was used for the open-eye experiment, with the following modification aimed to minimize the effect of blinks occurring intermittently in open-eye conditions. Frames that included blinks were excluded, and the calculated brightness was replaced by the linear extrapolation of the brightness values before and after the blinks. In cases where the blinks were at the start or end of the analysis time frame and only if the brightness reached a plateau level, a constant value with the value of the last/first nonblink frame was used to replace the blink brightness values. A total of 4 PLR events out of 31 (12.9%) were excluded from the analysis due to excessive blinking or blinks that did not enable an accurate linear extrapolation. At least three PLR events per configuration for each participant were included in the analysis after this exclusion.

#### 2.4.3 Closed-eye detection of pupil position

**Fig. 2:**
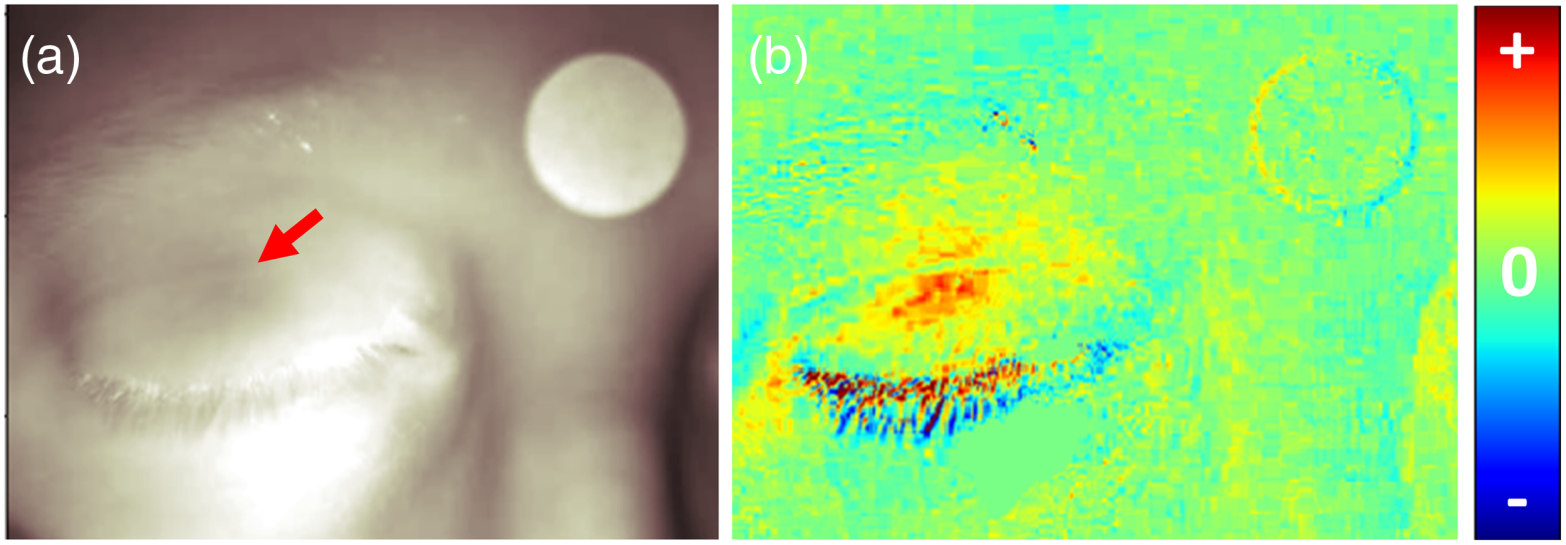
Identifying the pupil position in closed-eye images. (a) Original SWIR image of the participant’s face (captured with the crossed polarizer configuration). The light reflected from the iris can be seen as a dark region in the eyelid (marked by the red arrow). (b) Brightness difference image between the average SWIR brightness image during the 2 s before the white screen illumination and the image captured 1 s after the start of the white screen illumination. Red pixels in the eye reveal the area in which brightness increased, corresponding to where the pupil constricted. Some movement can also be seen around the eyelashes. The brightness scale can be adjusted to exclude changes outside the eyelid area.

In closed-eye experiments, especially when the participant is not looking forward, it is difficult to determine the position of the pupil. To identify the estimated position of the pupil, we used two visual aids. The first is the SWIR image of the participants’ eyes. As mentioned previously,^28^ it is possible to see the reflection of light from the central iris and pupil as a dark circle on the eyelid [Fig. 2(a)]. In addition, we used an image of the differences in the pixel intensity between the average intensity of 2 s baseline before the appearance of the light stimulus and 1 s after the stimulus onset (approximately the maximal change). Due to the short interval, the main change in the image is expected to be a result of the pupil size changes [Fig. 2(b)]. Occasionally, minimal facial movements may also be observed and can be used to assess participant movement. The diameter of the fixed circle average brightness calculation was set at 50 pixels, based on the most affected regions in these images, and slightly smaller than the size of the iris.

#### 2.4.4 Statistical analysis

Statistical analysis was conducted in R (version 4.5.1; R Core Team 2025).^44^ A linear mixed-effects model^45^^,^^46^ was used to evaluate the effect of polarization configuration on the maximal brightness changes and the SNR. Participant identity was included as a random intercept to account for repeated measurements within individuals. Polarization configuration was modeled as a fixed effect to assess its impact on the outcome. *Post hoc* pairwise comparisons among polarization configurations were performed using Tukey-adjusted tests via the multcomp package^47^ (glht()), with adjusted p-values computed using the single-step method.

## 3 Results

To improve gaze and pupil monitoring through closed eyelids, we first investigated the utility of adding polarizers to our optical setup to eliminate glare in SWIR images. Healthy participants sat, whereas their head rested on a dedicated chinrest and participated in experiments, where brief (2 s) light stimuli were presented every 20 s on a computer screen (Methods) to induce PLR, as described previously.^28^

### 3.1 Preliminary Experiments

**Fig. 3:**
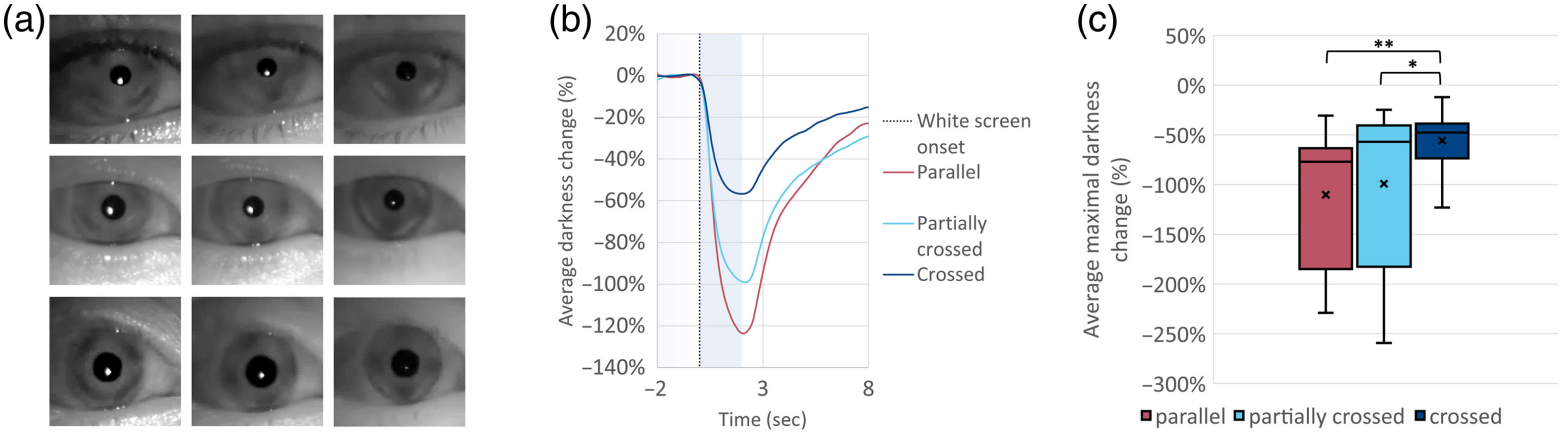
Open-eye experiment. (a) Images of participant eyes at the fully constricted state for all three polarizer configurations (left to right: parallel, partially crossed, and crossed orientations). The contrast between the iris and the pupil appears to be higher for the parallel configuration. (b) Grand average of all PLR events for each polarizer configuration (n=18 to 24 for each polarization configuration). The 2 s duration of the white screen trigger event is marked by the light blue background. (c) Comparison of maximal average darkness changes, revealing a significantly higher change for the parallel configuration compared with the crossed configuration (p=<0.001).

A preliminary open-eye experiment was conducted on three participants (female in her 40s, blue eyes; female in her 30s, green eyes; male in his 20s, brown eyes) to quantify the observed changes in the brightness of open eyes during PLR response. Each experiment included at least three PLR events for each of the three different polarizer configurations studied. All the visual stimuli induced PLR responses for all participants. Figure 3(a) shows images of the eyes of all participants for the different polarizer configurations at the fully constricted pupil states. Noticeably, for all the participants, the reflection from the skin around the eyes is higher in the parallel configuration. In addition, the iris appears darker for the crossed-orientation polarization compared with the partially crossed and parallel orientation polarizations. As the pupil is always black in SWIR images, the brighter iris appearance led to a higher contrast between the pupil and the iris in the parallel orientation (i.e., a more visible pupil). The visual pattern within the iris seems to be reversed between the parallel and the crossed configurations. This observation (more visible pupil in parallel orientation in open-eye settings) was supported by quantitative brightness changes calculated using the fixed circle approach across all PLR trials: changes were larger in amplitude in the parallel configuration throughout the PLR response [Fig. 3(b)].

We evaluated the effects of polarization configuration on both the maximal brightness change and the SNR using linear mixed-effects models with random intercepts for subjects. Maximum brightness change, expressed as a proportion of baseline, was significantly higher in the parallel configuration (M=1.09) than in the crossed (M=0.55; estimate = 0.600, p<0.001) and marginally higher than in the partially crossed configuration (M=0.98; estimate = 0.215, p=0.078). The partially crossed configuration also produced significantly greater brightness than the crossed configuration (estimate = 0.385, p=<0.001). The distribution is shown in Fig. 3(c).

For SNR, the parallel configuration again yielded the highest values (M=220), with marginally higher SNR compared with both crossed (M=74.7; estimate = 157.8, p=0.093) and partially crossed (M=65.4; estimate = 175.9, p=0.064) configurations. No significant difference was observed between the crossed and partially crossed configurations (p=0.973).

These findings suggest that, in open-eye settings, although both the parallel and partially crossed configurations enhanced brightness relative to the crossed configuration, only the parallel configuration provided an advantage in signal quality.

**Fig. 4:**
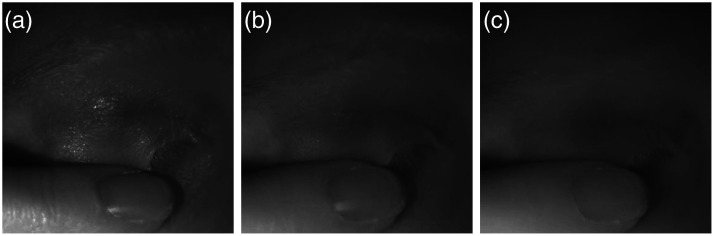
Closed-eye images imaged using different polarizer configurations. (a) Parallel configuration, (b) partially-crossed configuration, and (c) crossed configuration.

An opposite trend was observed when imaging closed eyes. Figure 4 shows an example of SWIR images of the same participant imaged with the three polarizer configurations. As expected, the specular reflectance from the surface of the eyelid skin was reduced with the crossed polarizer orientation. A similar reduction can also be seen in the skin and nail of the finger holding the eyelid. As these images demonstrate, there is a trade-off between better pupil contrast in the parallel configuration and the reduced noise due to the specular reflection in the crossed configuration.

### 3.2 Forward-Fixated PLR in Closed-Eye Setting

**Fig. 5:**
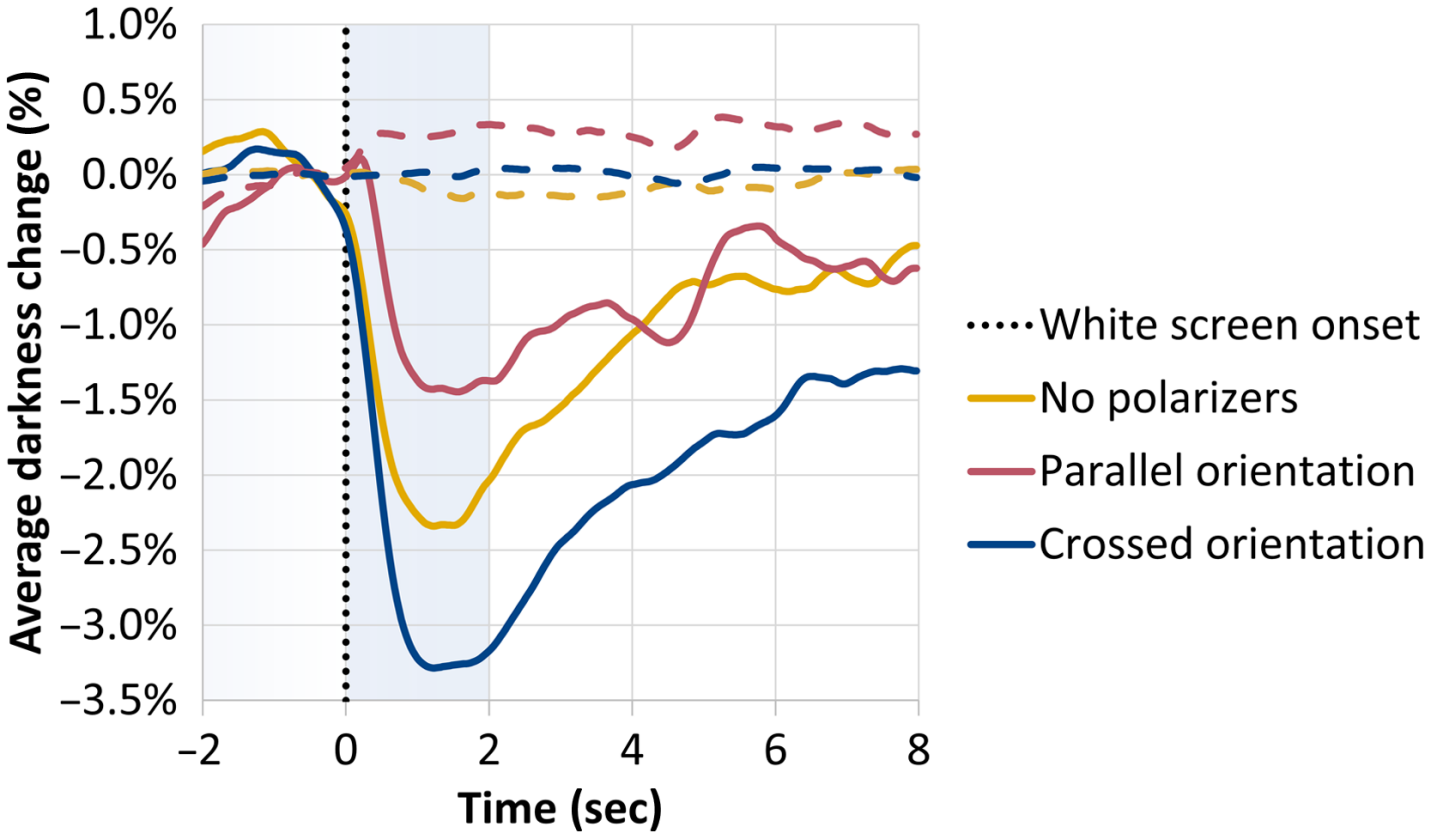
Grand average of the PLR events in the main closed-eye experiment. Continuous lines: average darkness change for the pupil area (n=120 trials for each configuration). Dashed lines: average darkness change for the forehead control area (n=60 trials for each configuration). Orange, no polarizers; red, parallel polarizer orientation; and blue, crossed polarizer orientation.

Three polarizer configurations (parallel and crossed polarizer orientation, and the comparative no-polarizer configuration, as described in Sec. 2) were used to capture PLR events in six participants (ages: 26 to 41, four females and two males). The experiments included 10 PLR events for each participant, analyzed separately for each eye, as described above. Figure 5 displays the grand average of all PLR responses for all participants at each configuration (n=120 trials: 6 participants × 2 eyes × 10 PLR events).

The crossed orientation configuration yielded the highest mean brightness change (M=0.0171), significantly greater than both the no-polarizer configuration (M=0.0106; estimate = 0.00644, p=0.035) and the parallel configuration (M=0.00580; estimate = 0.01126, p<0.0001). The difference between the parallel and no-polarizer configurations was not statistically significant (p=0.152).

SNR followed a similar trend, with the highest value observed in the crossed configuration (M=13.6), which was significantly greater than in the no-polarizer (M=9.79; estimate = 3.81, p=0.0015) and parallel (M=7.63; estimate = 5.97, p<0.001) configurations. No significant difference was found between the parallel and no-polarizer conditions (estimate = –2.16, p=0.118).

Together, these results show that in the closed-eye experiment, the crossed polarization configuration enhanced both brightness and signal quality compared with the no polarization or parallel orientation configurations.

### 3.3 PLR with Varying Gaze Directions in Closed-Eye Setting

**Fig. 6:**
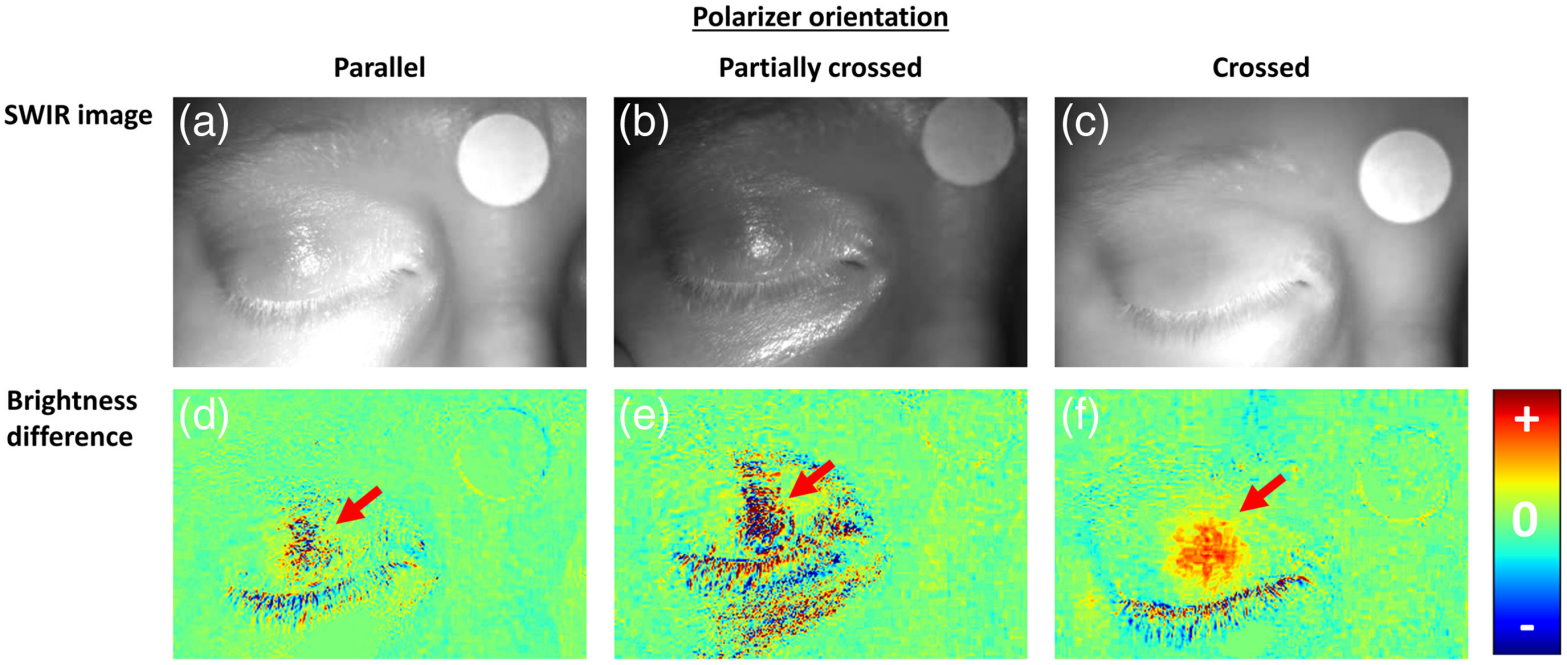
Comparing polarizer configuration to estimate pupil position in closed-eye settings. Brightness difference images for the different configurations, demonstrating the added value of the polarizers in removing the glare from the eyelids and revealing the pupil position (marked by arrows) more clearly. Columns represent (a) parallel configuration, (b) partially-crossed configuration, and (c) crossed configuration. (d)–(f) The brightness difference in images obtained before and after brief light stimuli was presented.

The same polarizer configurations were used to image PLR events when the participants were instructed to direct their gaze to different directions (Methods). The brightness difference and the SWIR images were used to identify the pupil for each PLR event separately, as described in Fig. 2. The crossed orientation configuration enabled better identification of pupil position as the specular reflection was very sensitive to small movements, leading to a more pronounced effect in the brightness difference images (Fig. 6).

**Fig. 7:**
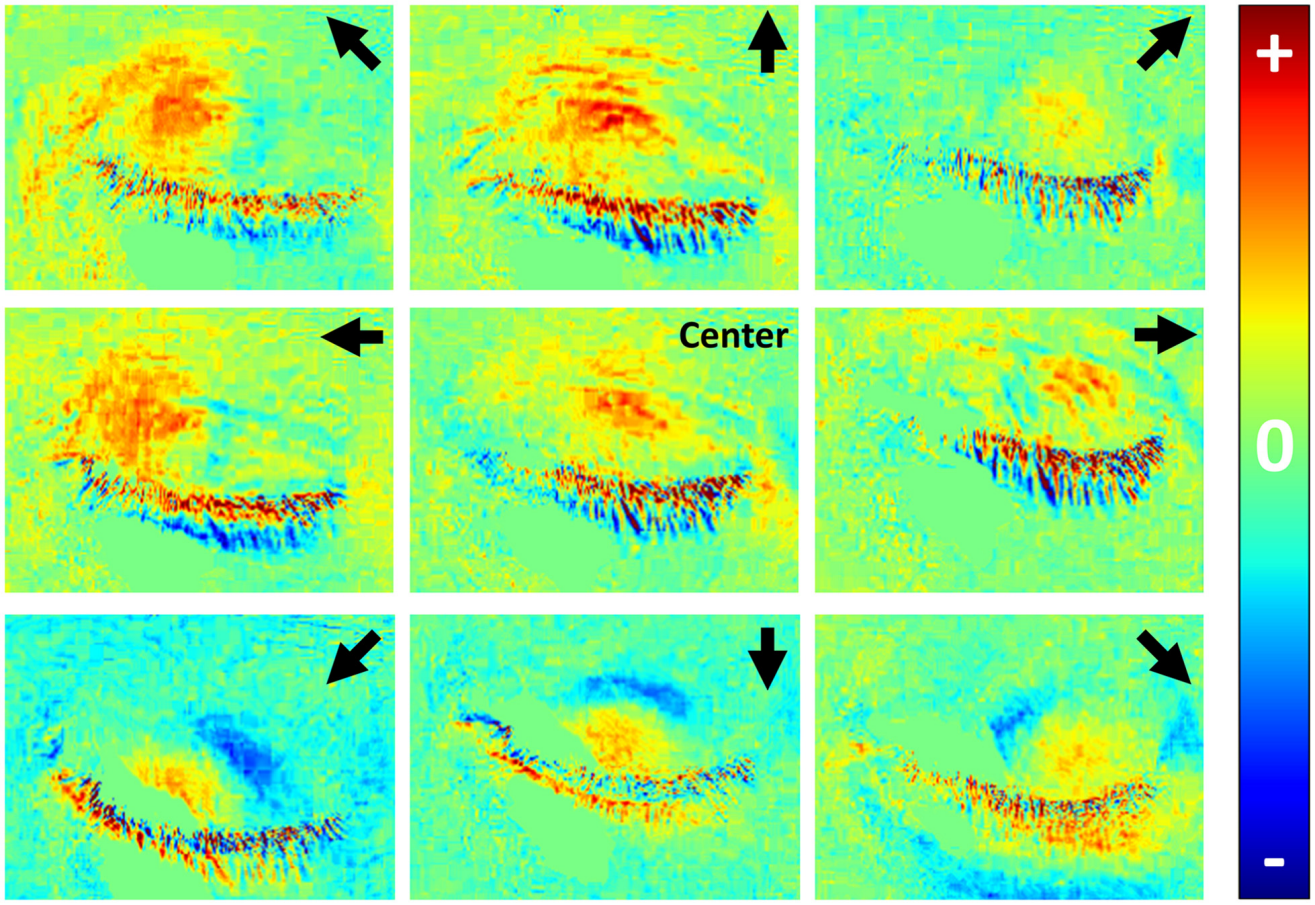
Imaging PLR events for varying gaze directions: single participant outcomes. SWIR subtraction images for nine different gaze directions. The red regions reveal areas in which the brightness increased, suggesting pupil constriction, which guided the positioning of the “fixed circle” region of interest for subsequent analysis. The images are organized according to the instructed gaze direction (marked by black arrows or “center” at the top right corners of each image).

Figure 7 displays the pupil identification analysis, for all nine gaze directions, in a representative individual for the right eye only. Pupil position could be identified in each gaze direction to guide the positioning of the “fixed circle” region-of-interest for analysis of PLR dynamics. As can be seen, the crossed orientation allowed the identification of the typical PLR responses in most gaze directions, even when applied to single trials, without the need to average over multiple events.

**Fig. 8:**
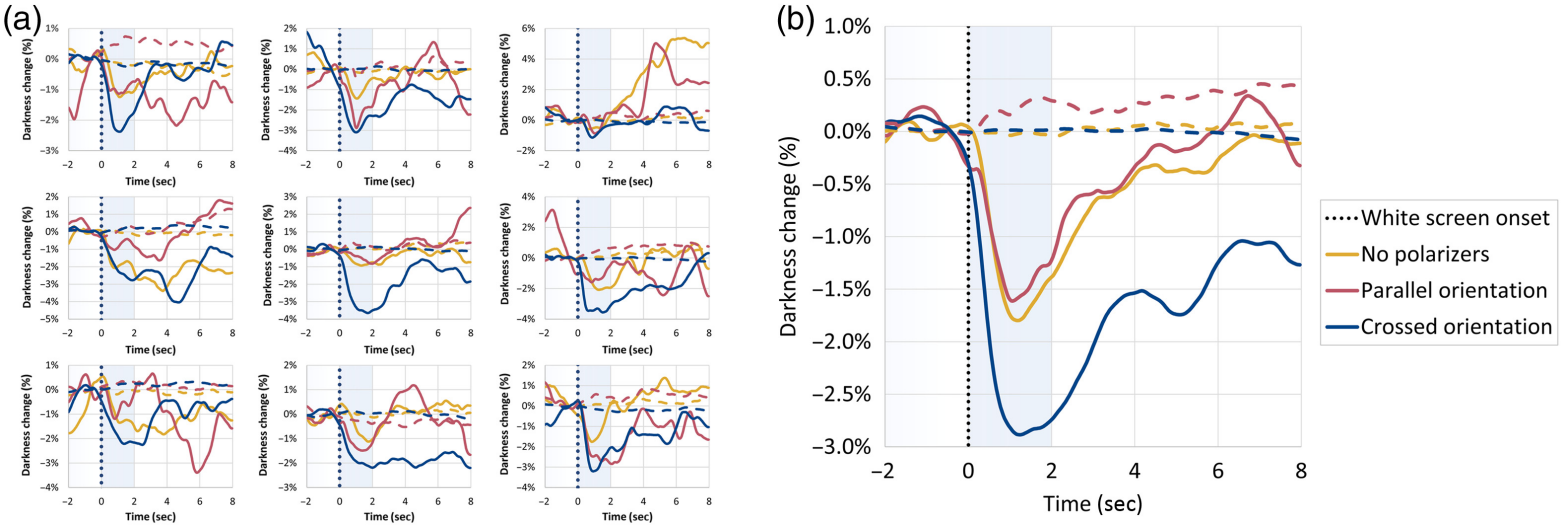
Imaging PLR events for varying gaze directions: grand average on all participants. (a) Darkness changes following the PLR events for each position, at each polarizer configuration (n=12 for each curve). (b) Grand average over all positions (n=108 for each configuration).

Next, we proceeded to quantitative analysis across the entire dataset. Figure 8 displays the outcomes of this experiment, averaged for both eyes, across all participants, and across all trials (n=108 PLR events for each polarizer configuration). The typical PLR response curves can be seen for most configurations and positions [Fig. 8(a)] and were most prominent in the crossed orientation. Some gaze directions showed poorer results. It should be noted that some of the participants commented that it was harder to maintain a fixed stare direction in some of the gaze directions (mostly the top-left and top-right directions). In addition, the eyelashes added noise to the analysis that was more evident in the bottom gaze directions. Despite these challenges, the grand averages of the responses for each of the configurations, over all gaze directions [Fig. 8(b)], display a very clear PLR response for all polarizer configurations. The crossed polarizer configuration produced the highest mean brightness change (M=0.0146), significantly greater than both the no-polarizer (M=0.00508; estimate = 0.00957, p=0.0079) and parallel (M=0.00433; estimate = 0.01031, p=0.0036) configurations. No significant difference was observed between the parallel and no-polarizer configurations (Estimate = –0.00075, p=0.97).

Similarly, SNR was highest in the crossed polarizer configuration (M=12.1), with significantly higher values than both the no-polarizer (M=6.90; estimate = 5.19, p<0.001) and parallel (M=5.78; estimate = 6.31, p<0.0001) configurations. Again, no significant difference emerged between the parallel and no-polarizer configurations (estimate = –1.12, p=0.66).

The forehead region in the parallel configuration was relatively noisier than for the other configurations, further demonstrating the problematic nature of specular reflection noise in this configuration. These findings further support the conclusion that crossed polarization markedly enhances both image brightness and signal quality in the closed-eye settings, whereas the parallel configuration does not confer improvements over the control configuration without polarizers.

## 4 Discussion

Closed-eye pupillometry has a big potential to impact multiple medical and scientific fields. In this study, we enhance the SWIR-based imaging method by introducing several key improvements over our original proof-of-concept report.^28^ Specifically, we incorporate polarizers into the optical setup, develop software tools capable of tracking pupil size despite positional changes, and perform imaging with both eyes naturally closed (without manually stretching the eyelid). These refinements enable imaging under conditions that more closely resemble clinical scenarios. Despite the added challenges of imaging through thicker, naturally closed eyelids (in previous experiments, the eyelid of the closed eye was stretched by placing a finger on it) and the uncertainty of pupil position, the maximal PLR amplitude averaged ∼2%, consistent with our previous findings.

The closed-eye experiments demonstrate a greater change in the PLR response when using the crossed polarizer configuration compared with the control setup without polarizers, mainly in the varying gaze direction experiment. This improvement is due to the reduced glare and the consequent improved pupil position estimation enabled by the cleaner image. This highlights the broader utility of polarization in biomedical imaging. By selectively filtering light based on its polarization state, the use of polarizers can enhance contrast, suppress surface reflections, and isolate subsurface scattering—features particularly beneficial for imaging through turbid or layered tissues such as skin or eyelids. Polarization-based techniques are increasingly used in various biomedical applications. For example, in dermatology, polarization imaging can improve visualization of vascular structures and pigmented lesions by filtering out surface glare and enhancing contrast in melanin-rich tissues.^48^ In neurosurgery, polarized-light imaging can be used to identify structures associated with cancerous tissue and assist in the detection of tumor borders.^49^ Similarly, a Mueller polarimetric endoscope provided multiple polarization-contrast images and demonstrated enhanced visualization of early epithelial cancers and clear tumor margins compared with standard endoscopy.^50^ Furthermore, polarization filtering can be implemented with minimal changes to existing hardware and without increasing illumination power, making it a low-cost, noninvasive strategy for enhancing signal fidelity in both research and clinical imaging systems.

The study also presented several improvements over the analysis algorithms described previously. The previous study was based on a more rigid experimental setup due to the need to establish the validity of this method and compare its pupil size and position estimations to a simultaneous open-eye control. Therefore, participants held one eye closed using their finger and kept the other eye open. As discussed in detail in the previous study, holding the eyelid does not represent clinically relevant scenarios, in which the eyes are naturally closed. In the current study, all closed-eye measurements were conducted with naturally closed eyes. This change necessitated accurate identification of the pupil position, which was performed using a combination of the raw SWIR images and brightness difference images. Although the eyelid skin is thicker when the eyelids are not stretched by the finger, the PLR response could be robustly identified in the new setting. Furthermore, removing the finger from the eye images reduced potential interferences due to finger movements and variability in the manner and extent of the eyelid skin stretching. In addition, in the last experiment of the current study, the participants were not instructed to gaze forward toward a fixed point, thereby moving closer to natural bedside clinical scenarios. Despite this change, PLR responses were identified in most of the gaze directions (pupil positions), suggesting that this method can be successfully applied to monitor pupil size even when eye movements occur freely. Due to the improved imaging and analysis, the maximal PLR amplitude was greater than that observed in the first study, despite the unstretched eyelids and the illumination intensity reduction due to the polarizers.

In this study, we observed an apparent reversal in the intensity trends across polarization states (crossed, partially crossed, and parallel) when comparing imaging of skin (where crossed polarizers are optimal) versus the open eye (where parallel polarizers are optimal). This discrepancy may arise from differences in the optical microstructure and scattering regimes between skin and the iris. The iris may exhibit more complex birefringent and scattering behavior due to its pigment content, layered structure, and variation in scattering particle sizes. Furthermore, previous studies have reported inter-subject variability in polarization responses at visible wavelengths, suggesting that individual anatomical differences could influence the optimal polarization configuration.^40^^,^^41^ Although initial data in our study hinted at such variability, the limited sample size precluded rigorous statistical analysis and warranted further investigation in future work.

Another possible explanation for the improved SNR and maximal brightness with the crossed orientation polarization when the eyelids were closed may arise from the internal structure of the eyelids that includes vertically aligned meibomian glands embedded within the tarsal plate.^51^ These elongated, lipid-rich glands may introduce anisotropic scattering that depends on both the orientation and polarization of the incident light, thus leading to the opposite trends observed between the open eyes and the closed eyes in the different polarizer configurations.

Several limitations still exist in the current study and should be acknowledged. First, although we eliminated the need to maintain a fixed gaze direction, the participants were still requested to maintain a fixed head position. At present, due to the nonuniform spatial illumination pattern of the LED, any movement could change the illumination on the eye and affect the results. Future studies should ensure a more uniform illumination to overcome this challenge. Including multiple light sources can also minimize shadows that can affect measurements. Beyond illumination, a tracking system can be implemented to allow for continuous imaging during head movements, as in the case of natural sleep. It should be noted, however, that applications for anesthesia or sedation monitoring could be implemented under the assumption that head movements are minimal. Software tools can also be used to partially overcome translation and stabilize eye position in the frame. Second, due to the long duration of each trial, only a limited number of polarizer configurations could be tested. Although the crossed configuration generally reduced glare, individual differences were observed. This suggests that the optimal configuration may vary among individuals, highlighting the potential value of personalization in future implementations. As the polarizers allow for better pupil position identification, it may be beneficial in some cases to use a polarizer configuration for the identification while observing the PLR signal through a configuration that does not include polarizers. Third, the study was conducted on a relatively small number of participants. To compensate for the relatively small sample size, 10 PLR events and both eyes were included in the analysis, bringing the total number of samples to 120 for each configuration.

Fourth, involuntary contractions of facial muscles, subtle movements, or transient changes in facial expression can introduce fluctuations in the optical signal that may reduce SNR in certain cases. Such fluctuations were observed in some of the experiments conducted. However, they were often smaller in magnitude than the PLR signal, and their duration was shorter than the several-second response of the PLR and therefore did not mask it. Future studies could further reduce sensitivity to such artifacts by employing more robust filtering strategies.

The current experimental setup employs an LED illuminating at 1100 nm, outside the most commonly utilized near-infrared optical window (650 to 950 nm) and within the second optical window of 1100 to 1350 nm.^52^ Imaging at this wavelength requires the use of a dedicated SWIR camera, which is more expensive than traditional NIR cameras due to differences in sensor technology. Nevertheless, this wavelength was selected because prior preliminary experiments with different LED wavelengths indicated that it produced a stronger signal. This advantage is likely related to the structure and optical properties of the eye: maximizing the contrast between iris reflection and the pupil is crucial for pupil identification while still minimizing attenuation through the eyelids increases the overall signal. Determining the optimal trade-off among these factors is complex and should be further investigated through both experimental measurements and simulations. A systematic evaluation could clarify whether other wavelengths in the NIR or SWIR range may improve the SNR. Furthermore, integrating multiple wavelengths may provide complementary information, and exploring this aspect represents a promising direction for future work.

Further research is also required to demonstrate the validity of this method for identifying changes in pupil size that are smaller in magnitude than the PLR and changes that occur in unknown times (not triggered by external stimuli). Such changes could occur due to the internal state, depth of anesthesia, pain, arousal, or cognitive processes. Next steps will include experiments to monitor smaller pupillary changes, such as in response to pain or auditory stimuli, and estimate the threshold of detection in different scenarios. In addition, future studies with large-scale data could also employ machine learning models to predict pupil size and position from the closed-eye images, along the lines of our previous study.^28^ Such models can potentially better overcome noise and variability compared with the analysis approach presented here.

## 5 Conclusion

Closed-eye pupillometry can benefit from the use of polarizers to reduce glare and improve pupil position identification. The use of the crossed polarizer orientation allows better identification of the pupil position during PLR events, which enables improved PLR detection and gaze direction in naturally closed eyes.

## Data Availability

Images of raw data cannot be shared due to privacy issues. Excel files created from the raw data will be available upon request. Code will be available as well upon request from the authors. We will guide those interested in how to retrieve it.
